# Diversity of the virome associated with alfalfa (*Medicago sativa* L.) in the U.S. Pacific Northwest

**DOI:** 10.1038/s41598-022-12802-4

**Published:** 2022-05-24

**Authors:** Lev G. Nemchinov, Brian M. Irish, Samuel Grinstead, Jonathan Shao, Paulo Vieira

**Affiliations:** 1 Molecular Plant Pathology Laboratory, USDA-ARS, Beltsville, MD 20705-2350 USA; 2 Plant Germplasm Introduction and Testing Research Unit, USDA-ARS, Prosser, WA 99164 USA; 3 National Germplasm Recourses Laboratory, USDA-ARS, Beltsville, MD 20705-2350 USA; 4 Mycology and Nematology Genetic Diversity and Biology Laboratory, USDA-ARS, Beltsville, MD 20705-2350 USA

**Keywords:** Virology, Plant sciences

## Abstract

Alfalfa (*Medicago sativa* L.) is one of the most extensively cultivated forage legumes in the world. It is currently the third most valuable field crop in the United States with an estimated value of over $9.3 billion. Alfalfa productivity is limited by various infectious diseases that can reduce forage yield and quality and shorten stand life. The crop can frequently be infected with a diverse array of pathogens and other organisms that have distinct life cycles, biology, and mode of action. Among them are many coinfecting viruses, that greatly contribute to the heterogeneity of within-host pathogenic communities, representing a ubiquitous and abundant background for all other host–pathogen interactions. Regrettably, the impact of viral diseases, their role in alfalfa health and involvement in the severity of multi-pathogen infections are often underestimated and not well understood. As high-throughput sequencing approaches have been developed, opportunities to delve into these complex interactions can be realized. In this work, we have characterized a diversity of viral populations in several commercial alfalfa production fields located in the U.S. Pacific Northwest. At least 45 distinct viruses have been identified in all alfalfa samples. Among them some were known to infect the crop prior to this study, and others were designated as emerging, novel and viruses integrated into the alfalfa genome. Known viruses included alfalfa mosaic virus, pea streak virus and bean leafroll virus, while among emerging and novel agents were alfalfa virus S, cherry virus Trakiya, several rhabdoviruses and others. Additional biological and impact studies will be needed to determine if newly identified viruses, especially those that have not been reported from alfalfa before, should be considered pathogens of this crop.

## Introduction

Alfalfa (*Medicago sativa* L.) is a major forage crop worldwide and the third most valuable field crop in the United States with an estimated value of over $9.3 billion^1^. Viral infections of alfalfa are widespread in major cultivation areas. Alfalfa may also serve as a natural reservoir for dissemination of viruses to other agriculturally important crops. Although several economically important viral diseases infecting alfalfa have been previously described, novel viruses continue to be discovered at accelerated rates thanks to the advent of high-throughput sequencing (HTS) technologies^1–9^. In the United States, among the major alfalfa-infecting viruses are alfalfa mosaic virus (AMV), bean leaf roll virus (BLRV), pea streak virus (PeSV) and others^10^. The makeup of the alfalfa virome continues to expand to include new and emergent species, different geographic locations, and previously unknown adaptations of viruses to alfalfa as a new host^1^.

In agricultural production settings alfalfa plants are coinfected with a substantial number of different viruses, including well-known species, newly discovered pathogens and viruses formerly described in other hosts^1,5,6,8,11^. The extent of mixed viral infections in alfalfa, based on the HTS-derived findings, is remarkable and can reach many co-infecting viruses in a single plant. In many if not all cases they are accompanied by non-viral infections^11,12^. Therefore, it is increasingly important to alfalfa pathology and plant pathology field in general to broaden studies of single disease-causing biological organisms by research on multi-pathogenic infections and their impact on plant health.

A comprehensive survey of alfalfa varieties grown in the U.S. is needed to identify, characterize, and prevent the spread of novel and emerging viruses and to avoid any related yield losses. In this work, we continue to explore a diversity of plant viruses infecting alfalfa, focusing on growing regions in the U.S. Pacific Northwest. The study is encompassing results of HTS screening of commercial and research alfalfa fields that occurred in two consecutive years, 2019–2020.

## Results

### Symptomatology

**Figure 1:**
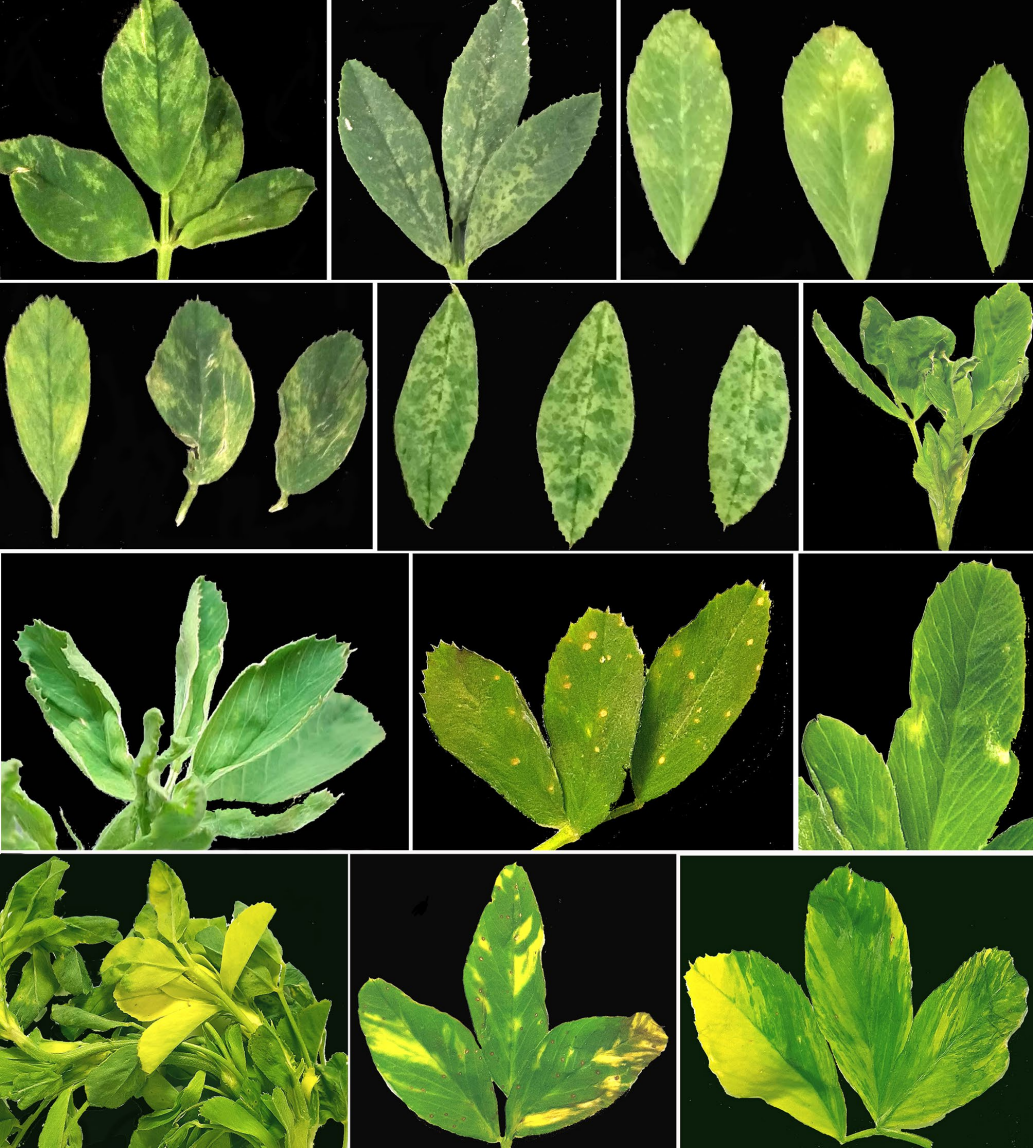
Symptoms observed in alfalfa samples collected from production fields in the U.S. Pacific Northwest in 2019/2020.

While many of the samples from alfalfa fields were visually asymptomatic, several plants had a range of symptoms documented in Fig. 1. These symptoms included typical virus-like symptomatology, such as leaf yellowing, mosaic, curling, distortion, mottling and necrotic spotting.

### RNA-seq data metrics

A total of 5,693,089,728 pair-end reads were generated from 91 cDNA libraries, averaging ~ 62,624,978 reads per library (Table S1). Overall, the data obtained were deemed sufficient for transcriptomic discovery and viral genome reconstruction from alfalfa field samples.

### Virus identification

Each of the analyzed samples had on average 12 different viruses (varying from five to 20), thus confirming a complexity of co-infections (Table S2). A total of 45 distinct viruses have been identified in all alfalfa samples (Table 1, Table S2). Among them ten were viruses previously reported and known to cause diseases in alfalfa. The remaining 35 were assigned to (a) emerging and known viruses not reported in alfalfa; (b) viruses that had no known record in the U.S.; (c) tentatively novel species; (d) pararetroviruses integrated in alfalfa genome; and (e) contaminant viruses that are likely originated from hosts other than alfalfa.

**Table 1:** A list of viruses identified based on RNA sequencing data in alfalfa samples collected from production fields in the U.S. Pacific Northwest in 2019/2020.

Virus names based on top BLAST hits	Proposed taxonomy	Known to infect alfalfa	%, presence
**Alfalfa latent virus**	Betaflexiviridae, Carlavirus	Y	13
**Alfalfa mosaic virus**	Bromoviridae, Alfamovirus	Y	85
**Alfalfa virus S**	Alphaflexiviridae, Allexivirus	Y	21
Ancient Northwest Territories cripavirus	Dicistroviridae, Cripovirus	N	7
Aphis glycines virus 1	Unclassified Picornavirales	N	7
**Bean leafroll virus**	Tombusviridae; Luteovirus	Y	34
Bombus-associated virus Pic2	Unclassified Picornavirales	N	5
Bundaberg bee virus 8	Unclassified Picornavirales	N	5
Cherry virus Trakiya	Unclassified Picornavirales	N	7
Datura yellow vein nucleorhabdovirus	Rhabdoviridae, Nucleorhabdovirus	N	59
Diabrotica virgifera virgifera virus 2	Unclassified	N	2
Diaphorina citri picorna-like virus	unclassified Picornaviridae	N	2
Faecal-associated gemycircularvirus	Genomoviridae; Gemycircularvirus	N	2
Figwort mosaic virus	Caulimoviridae; Caulimovirus	N	47
Garlic yellow virus	Unclassified Betaflexiviridae	N	4
Hop latent virus	Betaflexiviridae, Carlavirus	N	7
Horseradish latent virus	Caulimoviridae, Caulimovirus	N	7
Hubei picorna-like virus	Unclassified Picornavirales	N	17
Hubei toti-like virus 2	Unclassified Riboviria	N	33
HVAC-associated RNA virus 1	unclassified picorna-like virus	N	5
Kilifi Virus	Unclassified Picornavirales	N	2
Kinkell virus	Unclassified Iflaviridae	N	2
La Jolla virus	Unclassified Iflaviridae	N	2
Lasius niger virus 1	Polycipiviridae; Sopolycivirus	N	2
**Lucerne transient streak virus**	Solemoviridae, Sobemovirus	Y	12
Luckshill virus	unclassified Riboviria	N	2
Maize associated rhabdovirus	Rhabdoviridae, Cytorhabdovirus	N	9
Maize sterile stunt virus	Rhabdoviridae, Cytorhabdovirus	N	12
Maize yellow striate virus	Rhabdoviridae, cytorhabdovirus	N	59
Maize-associated picornavirus	Unclassified Picornavirales	N	5
**Medicago sativa alphapartitivirus 1**	Partitiviridae, Alphapartitivirus	Y	93
**Medicago sativa alphapartitivirus 2**	Partitiviridae, Alphapartitivirus	Y	56
**Medicago sativa amalgavirus 1**	Amalgaviridae; unclassified	Y	72
**Medicago sativa deltapartitivirus 1**	Partitiviridae, Deltapartitivirus	Y	54
Nesidiocoris tenuis iflavirus 1	Unclassified Iflaviridae	N	2
**Pea streak virus**	Betaflexiviridae, Carlavirus	Y	89
Picorna-like virus, unknown	Unclassified Picornavirales	N	22
Potato virus X	Alphaflexiviridae, Potexvirus	N	12
Pyrus pyrifolia cryptic virus	Partitiviridae, Deltapartitivirus	N	52
River Liunaeg virus	Unclassified Riboviria	N	2
Rudbeckia flower distortion virus	Caulimoviridae	N	5
Solenopsis invicta virus 7	Unclassified	N	2
Soybean chlorotic mottle virus	Caulimoviridae; Soymovirus	N	100
Twyford virus	Iflaviridae; Iflavirus	N	2
Zhuye pepper nucleorhabdovirus	Rhabdoviridae, unclassified	N	55

Bold font: viruses known to infect alfalfa.

### Common alfalfa viruses

Common viruses known in alfalfa in the U.S. included alfalfa mosaic virus (AMV), pea streak virus (PeSV), bean leafroll virus (BLRV), Medicago sativa alphapartitiviruses 1 and 2 (MsAPV1 and MsAPV1), Medicago sativa deltapartitivirus 1 (MsDPV1) and Medicago sativa amalgavirus 1 (MsAV1). These viruses were detected in many samples, some of them (AMV, PeSV, and MsAPV1) infecting more than 90% of all the plant samples evaluated (Table 1). Judging from the similarity in sequenced portions of their genomes (Table S2), they appeared to represent common isolates across samples and sampled sites.

### Emerging and known viruses previously not reported in alfalfa

Recently described alfalfa virus S (AVS), which is believed to be an emerging and possibly seed-transmitted virus^5,8^ was found in 21% of the samples collected in 2019 (Table 1). This confirms its occurrence in multiple sampled sites, at least in the surveyed fields for that year in that area.

A viral sequence resembling cherry virus Trakiya (CVT)^13^ was not reported to infect alfalfa as a host prior to this work. CVT-alfalfa strain (CVT-A) had 93% of complete genome identity to the reported cherry strain (NC_040561; 88% coverage; E-value = 0.0). Since assembled contigs of the CVT-A covered a significant portion of the putative viral genome (Table S2), we have completed its sequence by multiple RT-PCRs with virus-specific primers and by using 5'/3' RACE (Table S3). The complete genome of the virus from alfalfa is available in GenBank under accession number OK181162.

The total length of CVT-A genome was found to be 8706 nt, excluding a poly(A) tail. Like the cherry strain, the genome of CVT-A encoded two open reading frames (ORFs) and contained non-coding regions (NCRs) at the 5′-end (1–314 nt), 3′- end (8614–8706, 93 nt) and an intergenic NCR (2697–2937 nt). ORF1 encoded the putative coat protein (CP) consisting of 794 deduced amino acids (aa) with a predicted MW of 87.8 kDa. It was only 60.9% identical to the CP of CVT cherry strain (YP_009551962.1; 97% coverage, E = 0.0). However, ORF2 of CVT-A, encoding the putative replicase protein and comprised of 1892 aa with MW 215 kDa, was 94% identical to the reference strain (YP_009551963.1; 99% coverage; E = 0.0).

**Figure 2:**
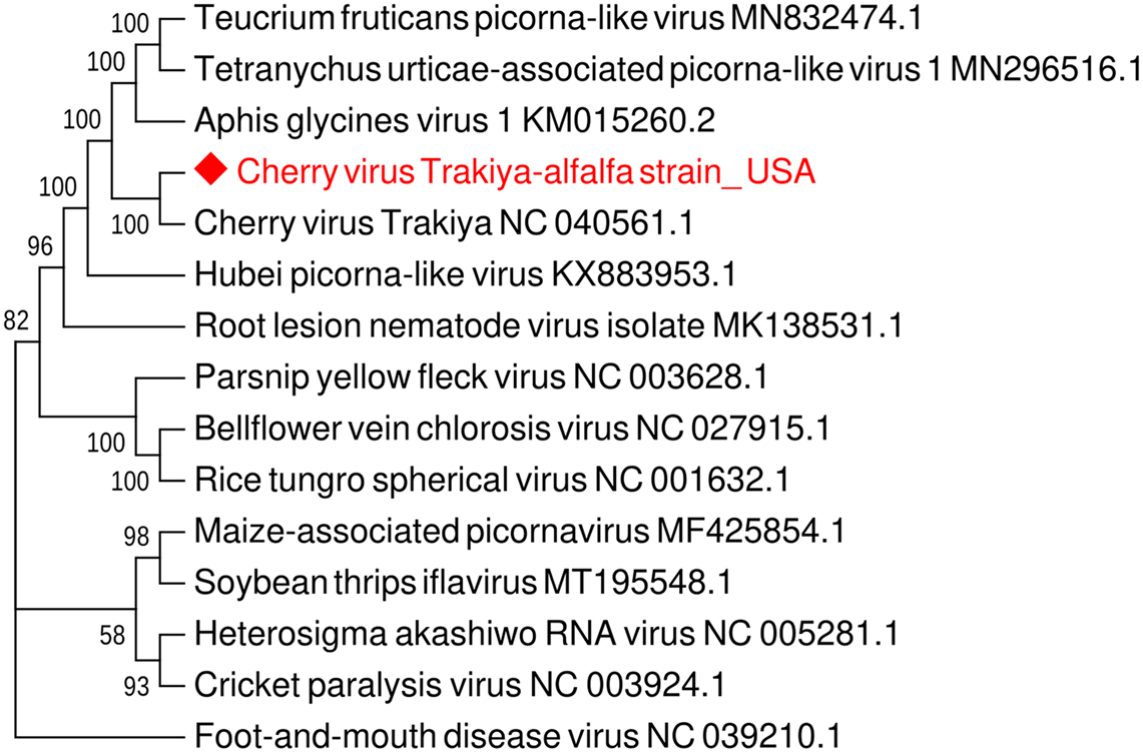
Phylogenetic relationship of cherry virus Trakiya, alfalfa strain, with other species of the order *Picornavirales*. The tree was deduced from the ClustalW alignment of complete nucleotide sequences and built using MEGA 7 software with Maximum Likelihood method based on the Tamura-Nei model and bootstrap analysis of 1000 replicates.

**Figure 3:**
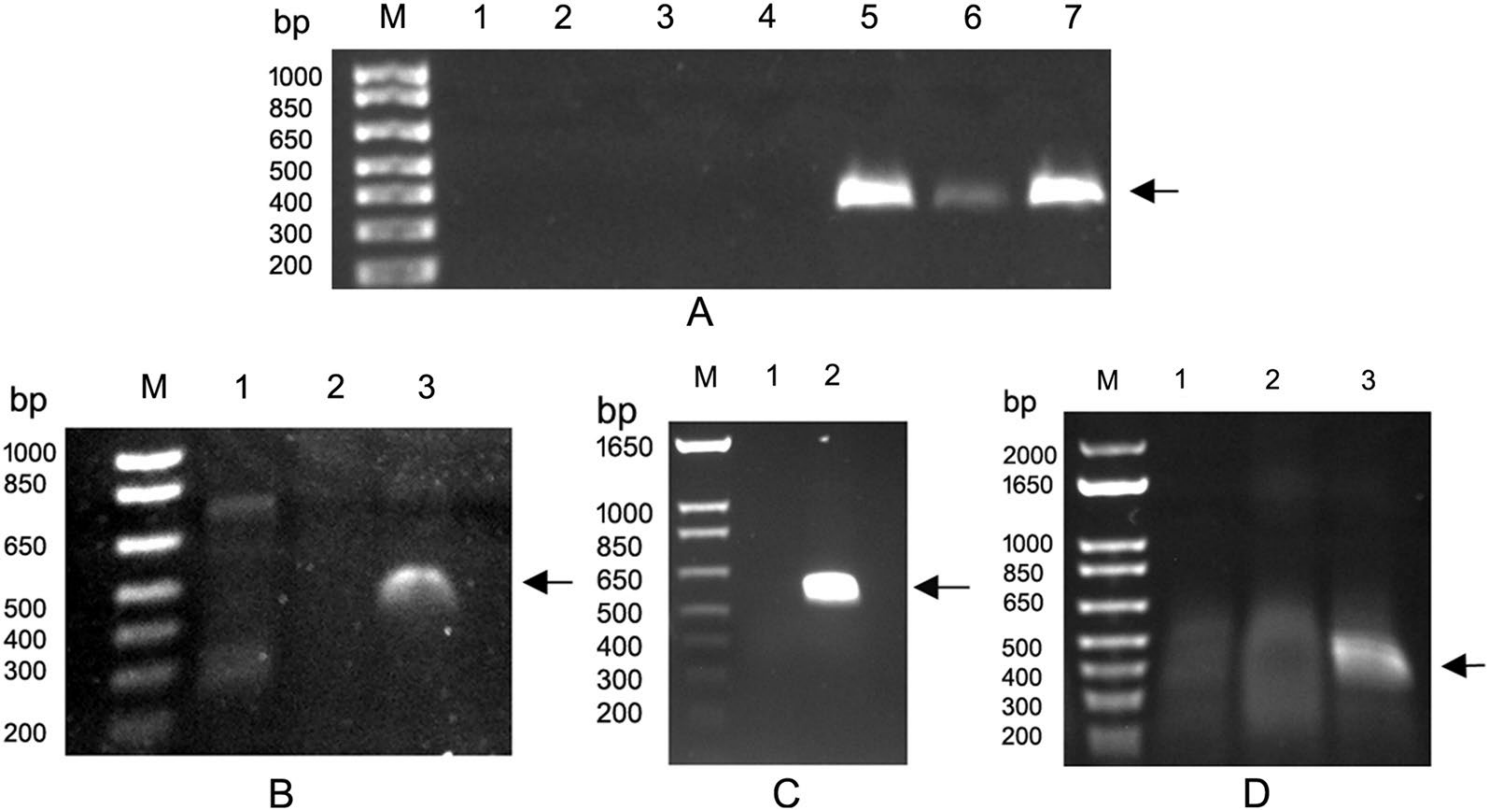
Detection of viruses identified from alfalfa samples collected from production fields in the U.S. Pacific Northwest in 2019/2020 using RT-PCR. (**A**) RT-PCR detection of cherry virus Trakiya, alfalfa strain. Lanes 1–4, negative PCR reactions; Lanes 5–7, PCR products amplified from different alfalfa leaves infected with CVT-A using primer pair LN882/883. (**B**) RT-PCR detection of hop latent virus using primers LN850/LN851. Lanes 1 and 2, negative samples. Lane 3, alfalfa sample infected with HLV. (**C**) RT-PCR detection of potato virus X using primers LN78/LN79. Lane 1, negative sample. Lane 2, PVX-infected sample. (**D**) RT-PCR detection of alfalfa nucleorhabdovirus 1 using primers LN846/LN847. Lane 1 and 2, negative reactions. Lane 3, a 437 bp RT-PCR product amplified from infected alfalfa leaves. M, 1 kb plus DNA ladder (Thermo Fisher Scientific, Waltman, MA USA). Arrows indicate amplified PCR products. Original full-length images of the gels are presented in the Supplementary Figure S1.

Phylogenetic analysis deduced from the alignment of the CVT-A with available complete nucleotide sequences of other viruses from the order *Picornavirales*, positioned CVT-A isolate in the same cluster with the cherry isolate (Fig. 2). It is therefore appearing that CVT-A, although quite diverse, represents a new strain of the same virus rather than a new picorna-like virus species infecting alfalfa. One of the primers pairs that was used to amplify missing segments and to complete genomic sequence of the CVT-A worked reliably in RT-PCR assays and can therefore be used for diagnostics of this novel virus strain (primers LN882/LN883, product size 353 bp), (Table S3), (Fig. 3A).

Two other viruses not previously reported in alfalfa but identified in this study by the data analysis pipeline included hop latent virus (HLV) and potato virus X (PVX). Sequenced portions of their genomes were highly identical to the reference viruses. Alfalfa-derived HLV contigs had on average 97–100% identity; while those that matched to the PVX contigs were 95–100% identical. Assembled HLV reads formed several short contigs; the longest of them was 926 nt and corresponded to the viral CP. The RT-PCR with several infected samples and primers specific for the nucleotide sequence of this HLV contig (LN850/LN851, Table S3) led to amplification of the product of the predicted size (473 bp). The 3' RACE with a sense HLV primer LN580 and the universal Takara Primer mix resulted in amplification of the complete CP gene and a 3' non-coding region including poly(A) tail (not shown). Sanger sequencing of the purified PCR products confirmed the identity of the virus as HLV (Fig. 3B). Assembled PVX reads, found in alfalfa plants from three different fields, represented all five ORFs of the virus: partial RNA-dependent RNA polymerase (RdRp) gene, triple gene block (TGB) 1–3, and CP gene, (Table S2). The ORFs shared 99–100% identity with the reference PVX genomes. The RT-PCR with PVX-specific primers (Table S3) led to amplification of the correct-size product (573 bp) from PVX-positive sample (Fig. 3C). Sanger sequencing of the purified PCR reactions confirmed the identity of the virus as PVX.

Despite the positive RT-PCR detection of these two viruses in alfalfa samples, an incidental contamination of the alfalfa plants rather than a true infection remains a strong possibility. When indicator species *Nicotiana benthamiana* plants were rub-inoculated with extracts from alfalfa samples in which the most of PVX reads were found, no characteristic PVX symptoms were observed. Indicator plants that had been rub-inoculated were RT-PCR-negative with PVX-specific primers (data not shown).

### Viruses not described in U.S. alfalfa

Viruses not known to occur in the US, but reported from other countries, included lucerne transient streak virus (LTSV)^14^ and Medicago sativa deltapartitivirus 1 (MsDPV 1)^15^. Although LTSV strain STN1 has been recently reported in the U.S.^16^, it was isolated from soybean thrips (*Neohydatothrips variabilis*) and not from naturally infected alfalfa plants. It thus cannot be considered an alfalfa isolate since LTSV naturally infects at least 18 species in four plant families^17,18^.

LTSV was found in 12% of tested samples originating from eight different alfalfa fields. The longest assembled LTSV contig (4257 nt) apparently represented a coding-complete genome of the US alfalfa isolate of LTSV (LTSVa-US). The top BLAST hits for this nucleotide sequence (97% identity, 99% query cover, E = 0.0) were genomic sequences of LTSV US isolate from thrips (MT224146.1) and LTSV-Can, a Canadian isolate of the virus (JQ782213.1). LTSVa-US encoded four ORFs, presumably corresponding to the movement protein (ORF1); viral polyprotein translating via − 1 ribosomal frameshifting into replicase-associated proteins (ORF2a and ORF2b); and CP (ORF4). The translated ORFs were 96–99% identical to the US isolate of the virus^16^.

**Figure 4:**
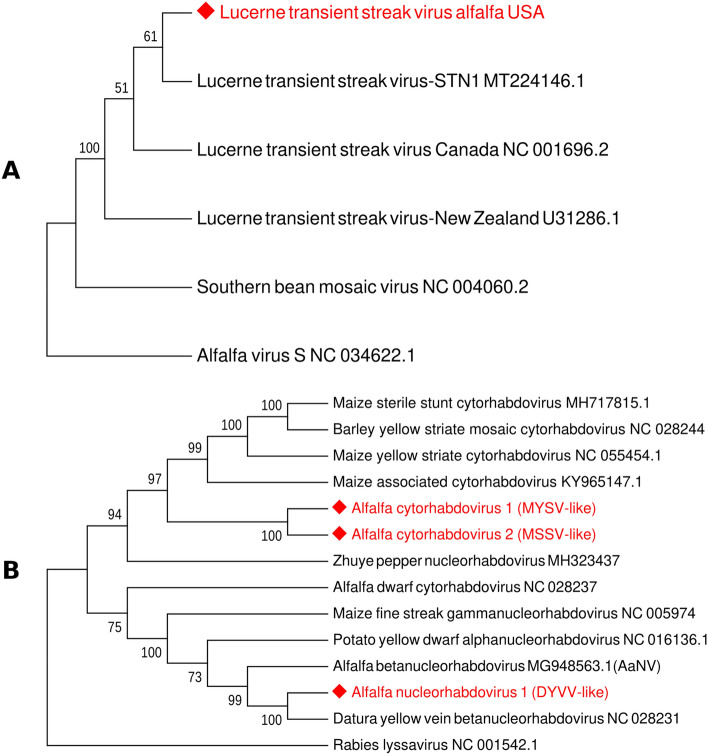
Phylogenetic analysis of lucerne transient streak virus (LTSV) and rhabdoviruses identified in alfalfa samples collected from production fields in the U.S. Pacific Northwest in 2019/2020. (**A**) Phylogenetic relationship of lucerne transient streak virus (LTSVa-US) with other known isolates of LTSV. The tree was deduced from the ClustalW alignment of LTSVa-US and complete nucleotide sequences of other LTSV isolates. Alfalfa virus S (NC 034622.1) was used as an outgroup. (**B**) The phylogenetic relationship between rhabdoviruses identified in this study and several classified or proposed members of the family *Rhabdoviridae*. The tree was deduced from the ClustalW alignment of the proposed new rhabdoviruses with the complete nucleotide sequences of known rhabdoviruses. Rabies lyssavirus (NC 001542.1) was used as an outgroup. The trees were built using MEGA 7 software with Maximum Likelihood method based on the Tamura-Nei model and bootstrap analysis of 1000 replicates.

Phylogenetic analysis, deduced from the alignment of the LTSVa-US with available complete nucleotide sequences of LTSV, placed LTSVa-US in the same cluster with the virus isolate from thrips (Fig. 4A). While relationship of the LTSVa-US to other isolates and to the *Southern bean mosaic virus*, a type species of the genus *Sobemovirus*, was evident, relatively low bootstrap values indicated its divergent status within the group. The nucleotide sequence of the LTSVa-US was submitted to GenBank under accession number OK181163.

RdRp of the MsDPV1 isolates was 99–100% identical at both nucleotide and amino acid levels to the previously reported isolates of the virus indicating a low genetic diversity among American isolates of MsDPV1 (Table S2).

### Tentatively novel species

Potentially novel species included rhabdo-, toti-, partiti, and picorna-like viruses. Top BLASTX hits were recorded for maize yellow striate cytorhabdovirus (MYSV; identity 31–44%, coverage 91%, E = 3e−36, N protein); datura yellow vein nucleorhabdovirus (DYVV; identity 52–76%, N protein); maize sterile stunt cytorhabdovirus (MSSV; identity 32–42%, N protein); maize-associated cytorhabdovirus (MaCV; identity 34–37%, putative N protein); zhuye pepper nucleorhabdovirus (ZPNRV; 64–75%; putative N protein), maize-associated picornavirus (identity 70–74%, CP); and horseradish latent virus (58–60%; polyprotein). Low identity and coverage levels indicated that these viruses are likely new species or at the very least new strains of the known pathogens (Table 2).

**Table 2:** Putative novel viruses identified in alfalfa samples collected from production fields in the U.S. Pacific Northwest in 2019/2020.

Contig ID	Top BLAST hit	Accession	%Identity	Provisional name	GenBank ID
23296	Datura yellow vein nucleorhabdovirus	KM823531	65	Alfalfa nucleorhabdovirus 1 (ANRV 1)	OK514705
7492	Maize yellow striate cytorhabdovirus	YP_010086409.1	31	Alfalfa cytorhabdovirus 1 (ACRV 1)	OK514706
75314	Maize sterile stunt cytorhabdovirus	QBJ27588.1	38	Alfalfa cytorhabdovirus 2 (ACRV 2)	OK514707
30828	Hubei toti-like virus 2	YP_009336495.1	45	Alfalfa-associated toti-like virus 1 (AATLV 1)	OK514708
18473	Ryboviria spp; Arma picornavirus GZ	QKN89031; QNJ34549	31–46	Alfalfa-associated picorna-like virus (AAPLV 1)	OK514709
49839	Picorna-like virus	QED21508.1	25	Alfalfa associated picorna-like virus (AAPLV 2)	N/A
67368	Pyrus pyrifolia cryptic virus	BBA20646.1	36	Alfalfa deltaparitivirus (ADPV)	OK514710

#### Alfalfa nucleorhabdovirus 1

The longest contig comprised 2646 nt and resembled nucleocapsid protein of DYVV. At the nucleotide level, it was 73% identical to DYVV (KM823531.1; coverage 31%; E = 5e−93). The largest translated ORF of the DYVV-like virus was 449 aa-long and only 65.7% identical to the nucleocapsid protein of the DYVV (YP_009176972.1). When this ORF was compared to the nucleocapsid protein of recently discovered alfalfa associated nucleorhabdovirus (AaNV^7^), the percent identity was 34.5%, indicating unlikely relationship between these two viruses. Provisionally, we named this virus alfalfa nucleorhabdovirus 1 (ANRV1). The longest contig homologous to ZPNRV (contig_2410, 1894 nt) was 98% identical to the ANRV1 and therefore most likely depicted the same virus. Using PCR primers designed according to the nucleotide sequence of the ANRV1 contig_23296 (LN846/LN847, Table S3), we were able to amplify a specific product of the predicted size (393 bp) from alfalfa samples (Fig. 3D). The product was sequenced and found to be identical to the ANRV 1.

#### Alfalfa cytorhabdovirus 1

The second longest contig had a top BLAST hit with MYSV (2444 nt) and contained one complete ORF 437 aa-long that was 31% identical (94% coverage; E = 5e−43) to the N protein of MYSV (YP_010086409.1) and 33% (94%; 4e−43) identical to the N protein of barley yellow striate mosaic cytorhabdovirus. At the nucleotide level, however, no significant similarity with other viruses was found, not counting short stretches of the sequence with a low coverage (2%). Despite the low levels of identity, these were sufficient to establish affiliation with the family *Rhabdoviridae*. We provisionally designated this new virus as alfalfa cytorhabdovirus 1 (ACRV1).

#### Alfalfa cytorhabdovirus 2

The third longest contig among those potentially belonging to the new viruses, consisted of 1903 nt and encoded a 260 aa-long fragment 38%, 37% and 35% identical to the nucleocapsid proteins of MSSV (QBJ27588.1), MYSV (YP_010086409.1), and MaCV (ARS22490.1), respectively. This virus was provisionally named alfalfa cytorhabdovirus 2 (ACRV2). Nucleotide sequence identities between partial segments of ACRV1 and ACRV2 was ~ 52%. Amino acid sequence identities between their translated ORFs, though, was ~ 84%, suggesting these could be different strains of the same virus.

To further clarify the relationship between all newly identified alfalfa rhabdoviruses, we performed phylogenetic analysis that also included classified and proposed members of the family *Rhabdoviridae* (Fig. 4B). Alfalfa cytorhabdoviruses ACRV1 and ACRV2 clustered together, branching to the known plant cytorhabdoviruses, while alfalfa nucleorhabdovirus (ANRV1) formed a group with DYVV, indicating their evolutionary relationship. Characteristically, previously discovered alfalfa rhabdoviruses, ADV^2^ and AaNV^7^, appeared to be divergent from the newly found pathogens.

Two other conditional novel viruses were represented by short contigs (< 500 nt; contig_257394 and contig_22223, Table S2), which did not allow their accurate identification. The top BLAST hits (nucleotides sequences) corresponded to the maize-associated picornavirus (MF425854.1; identity 71%, E-value = 3e−62; query cover 100%) and peanut chlorotic streak caulimovirus (U13988.1; identity 71%; E-value = 6e−25; query cover 77%), respectively. Translated portion of the contig contig_22223 had a top BLAST hit with horseradish latent caulimovirus.

#### Alfalfa-associated toti-like virus

**Figure 5:**
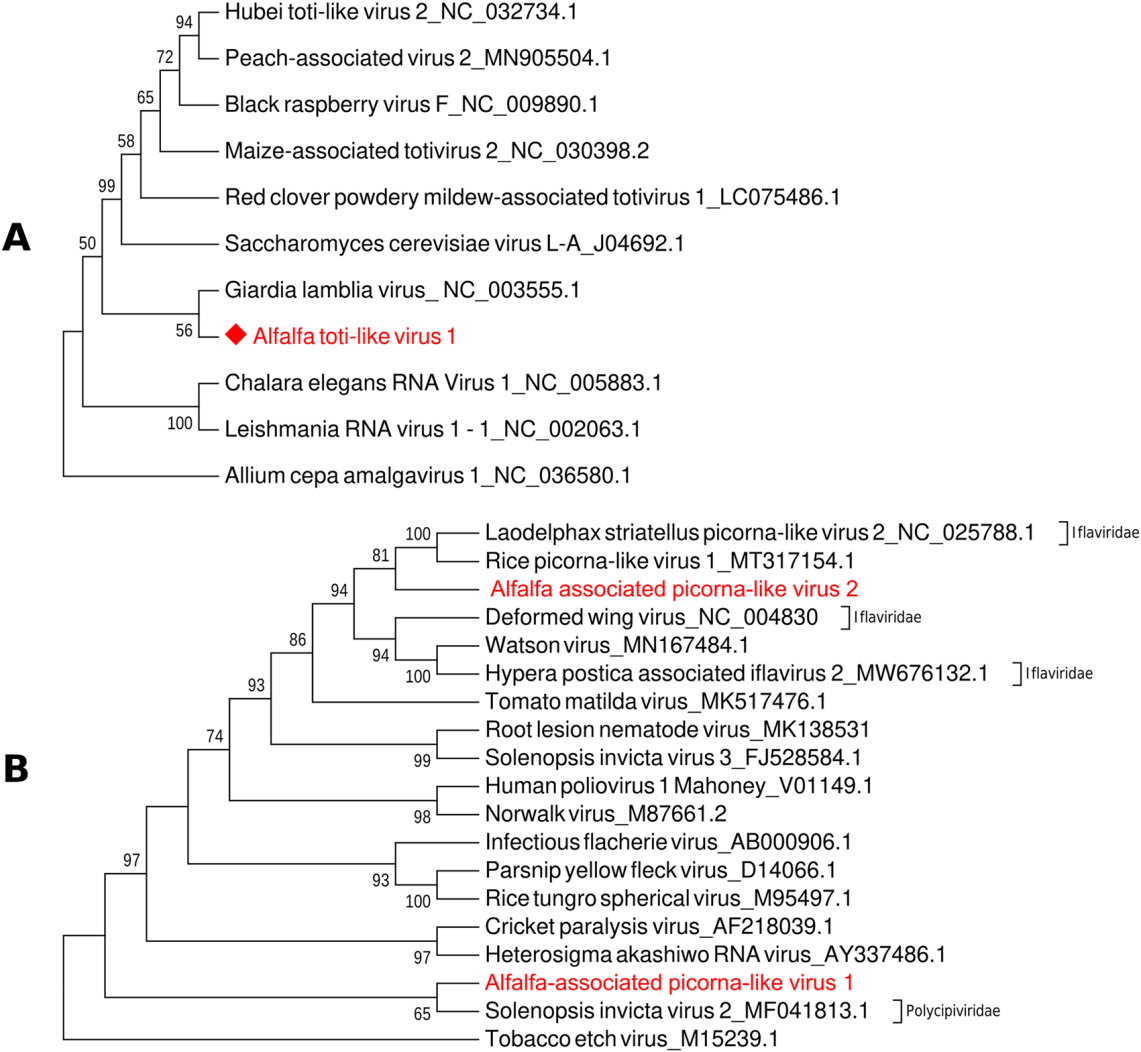
Phylogenetic analysis of the newly identified toti-like and picorna-like viruses found in alfalfa samples collected from production fields in the U.S. Pacific Northwest in 2019/2020. (**A**) The phylogenetic tree was deduced from the ClustalW alignment of the proposed alfalfa toti-like virus with the complete nucleotide sequences of the representative species and unclassified members of the family *Totiviridae*. Allium cepa amalgavirus 1_(NC_036580.1) was used as an outgroup. (**B**) The phylogenetic tree was deduced from the ClustalW alignment of the proposed alfalfa-associated picorna-like viruses AAPLV1 and AAPLV-2 with the complete nucleotide sequences of the selected members and unclassified viruses of the order *Picornavirales*. Tobacco etch virus_M15239.1 was used as an outgroup. The trees were built using MEGA 7 software with Maximum Likelihood method based on the Tamura-Nei model and bootstrap analysis of 1000 replicates.

Contig_30828 ORF (Table S1) was the longest one (1396 nt) comprising a sequence of an unclassified toti-like virus, a tentative member of the family *Totiviridae*, of single molecule linear dsRNA viruses. A translated portion of this contig had 45% identity (99% coverage; E-value = 1e−86) with a hypothetical protein of uncategorized Hubei toti-like virus 2 (YP_009336495.1). Nucleotide sequence search has not resulted in any hits. Although totiviruses are associated with latent infections of fungal or protozoan hosts^19^, among the top BLAST hits were also CP of several unclassified toti-like viruses apparently infecting higher plants: peach-associated virus 2 (QSV39137.1), black raspberry virus F (YP_001497150.1), and maize-associated totivirus 2 (YP_009259485.2). Phylogenetic analysis of the contig_30828 along with complete nucleotide sequences of the representative species and unclassified members of the family *Totiviridae* positioned it, albeit with poor bootstrap support, in the same cluster with *Giardia lamblia virus* (GLV), the prototype virus of the genus *Giardiavirus* that infects the parasitic protozoan *G. lamblia* (Fig. 5A). The cluster branched toward the *Saccharomyces cerevisiae virus L-A*, type species of the genus *Totivirus*. Based on the low identity values and inconclusive phylogenetic analysis, we propose that this contig could represent a fragment of the new virus, provisionally named alfalfa-associated toti-like virus (AATLV), although a possibility exists that this is a contaminant virus originating from a non-plant host.

#### Alfalfa-associated picorna-like virus 1

Several contigs, according to BLAST analysis, were associated with a group of different unclassified Hubei picorna-like viruses, reported *en masse* by Shi et al.^20^. The longest contig of 3387 nt translated into two proteins of 639 and 452 aa-long, with an intergenic region of 65 nt. The largest ORF had a low identity (31%) BLAST hit with a hypothetical protein of *Ryboviria* spp. (QKN89031.1), while the second ORF was paired with uncategorized Arma picornavirus GZ (QNJ34549.1; 46% identity). Nucleotide sequence search has not resulted in any significant hits with viral genomes. This virus was tentatively named alfalfa-associated picorna-like virus (AAPLV 1).

#### Alfalfa associated picorna-like virus 2

Many alfalfa samples collected in 2020 incorporated reads from an unknown picorna-like virus. Several overlapping contigs (contig_14119, contig_49839, contig_13431, contig_33544, and contig_19781) of this virus produced a 7277 nt-long sequence that translated into one polyprotein of 2412 aa. The ORF had best BLAST hits with Watson virus (QED21508.1) and Hypera postica-associated iflavirus 2 (HPAV, QUS52853.1). The former is an unclassified picornavirus reported from Australian fleas^21^ and the latter was recently found in larvae of alfalfa weevils (*Hypera postica*), a herbivorous pest feeding on alfalfa and other legumes^22^. As of today, the only known plant-infecting ifla-like virus has been identified from asymptomatic tomato plants^23^. However, the percent identity of the unknown picorna-like virus with the polyproteins of both Watson virus and HPAV was rather low (25–26%; 80% coverage), indicating that it could be an undescribed species originated from alfalfa or an ambiguous host. Phylogenetic analysis, encompassing the picorna-like virus, its top BLAST matches, and selected species from the order *Picornavirales* indicated its relationship with iflaviruses. The virus appeared only remotely connected to the AAPLV1 that clustered with Solenopsis invicta virus 2 from the family *Polycipiviridae* (Fig. 5B). Since, likewise AAPLV1, it was identified in RNA extracted from alfalfa samples without any visible contaminants, the virus, irrespectively of its exact origin, was provisionally named alfalfa associated picorna-like virus 2 (AAPLV 2).

#### Alfalfa deltaparitivirus

**Figure 6:**
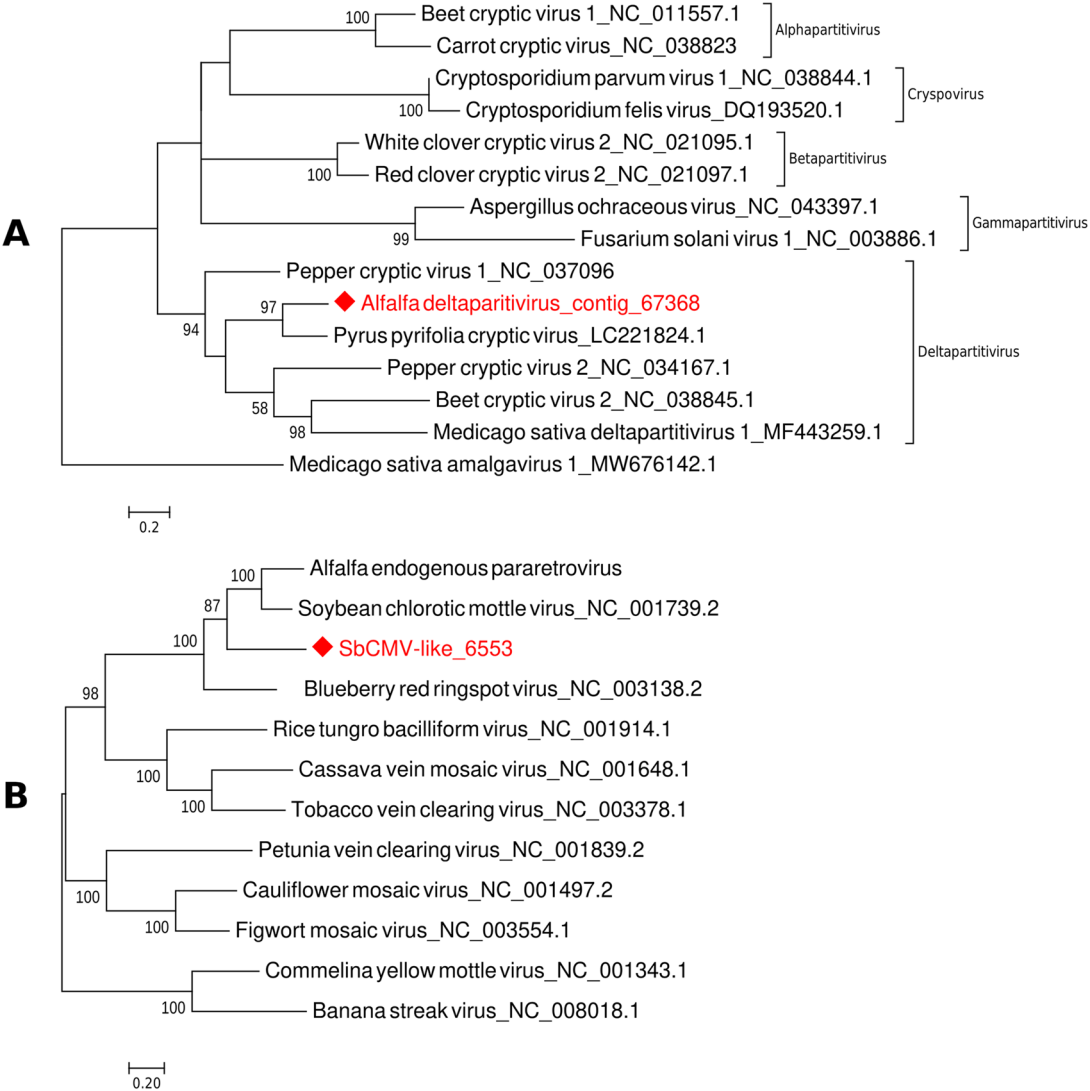
Phylogenetic analysis of the newly identified alfalfa deltaparitivirus (ADPV) found in alfalfa samples collected from production fields in the U.S. Pacific Northwest in 2019/2020, and endogenous virus resembling soybean chlorotic mottle virus (SbCMV-like). (**A**) The phylogenetic tree was deduced from the ClustalW alignment of the proposed ADPV with the complete nucleotide sequences of the representative members of the family *Partitiviridae*. Medicago sativa amalgavirus 1 (MW676142.1) was used as an outgroup. (**B**) The phylogenetic tree was deduced from the ClustalW alignment of the SbCMVL with the complete nucleotide sequences of the representative species from each genus of the family *Caulimoviridae.* The trees were built using MEGA 7 software with Maximum Likelihood method based on the Tamura-Nei model and bootstrap analysis of 1000 replicates.

More than half of the samples collected in 2019 contained nucleotide sequences that had BLAST hits with capsid protein of Pyrus pyrifolia cryptic virus (PpCV), a tentative member of the genus *Deltapartitivirus*, family *Partitiviridae*. The virus was first detected in Japanese pear^24^. Translated ORF (445 aa) of the longest contig (contig_67368; 1531 nt) had only 36% identity with PpCV (98% coverage, E-value = 1e−91), thus assuming that this could be a fragment of the new partitivirus infecting alfalfa. This contig presumably represented a complete nucleotide sequence of the RNA 2 segment of the virus, including poly (A) tail. Indeed, phylogenetic analysis clustered contig_67368 with PpCV, suggesting their relationship, as well as association with other deltapartitiviruses (Fig. 6A). The new virus was provisionally named alfalfa deltaparitivirus (ADPV). Interestingly, translated ORF of the ADPV was only 23% identical to the previously reported Medicago sativa deltaparitivirus 1 (MsDPV1)^15^. On the nucleotide level, their sequences were even less identical (10%), which was also reflected in their distant phylogenetic grouping within the family.

### Endogenous (integrated) viruses

All tested alfalfa samples (100%) had sequences homologous to soybean chlorotic mottle virus (SbCMV; nt identity ~ 66–68%) and 47% of the samples contained sequences homologous to figwort mosaic virus (FMV; nt identity % ~ 99–100%). While the SbCMV-like (SbCMVL) contigs derived from different samples assembled into nearly complete (~ 80%) genome of 6553 nt in length, FMV-like sequences were relatively short and assembled into a partial genomic fragment (Table S2). We have previously reported that two currently available alfalfa genomes, tetraploid^25^ and diploid (Cultivated Alfalfa at the Diploid Level; http://www.medicagohapmap.org/downloads/cadl), carry endogenous viral elements (EVEs) resembling SbCMV and FMV^26^ and that SbCMV EVEs may represent a new virus, provisionally named alfalfa endogenous pararetrovirus (AePV). In the phylogenetic tree, deduced from the alignment of the 6563 nt-long SbCMVL sequence with AePV and the complete nucleotide sequences of the representative species from the family *Caulimoviridae*, SbCMVL grouped in the same cluster with SbCMV and AePV (Fig. 6B). It is thus possible that SbCMVL sequences found in this study depict AePV.

**Figure 7:**
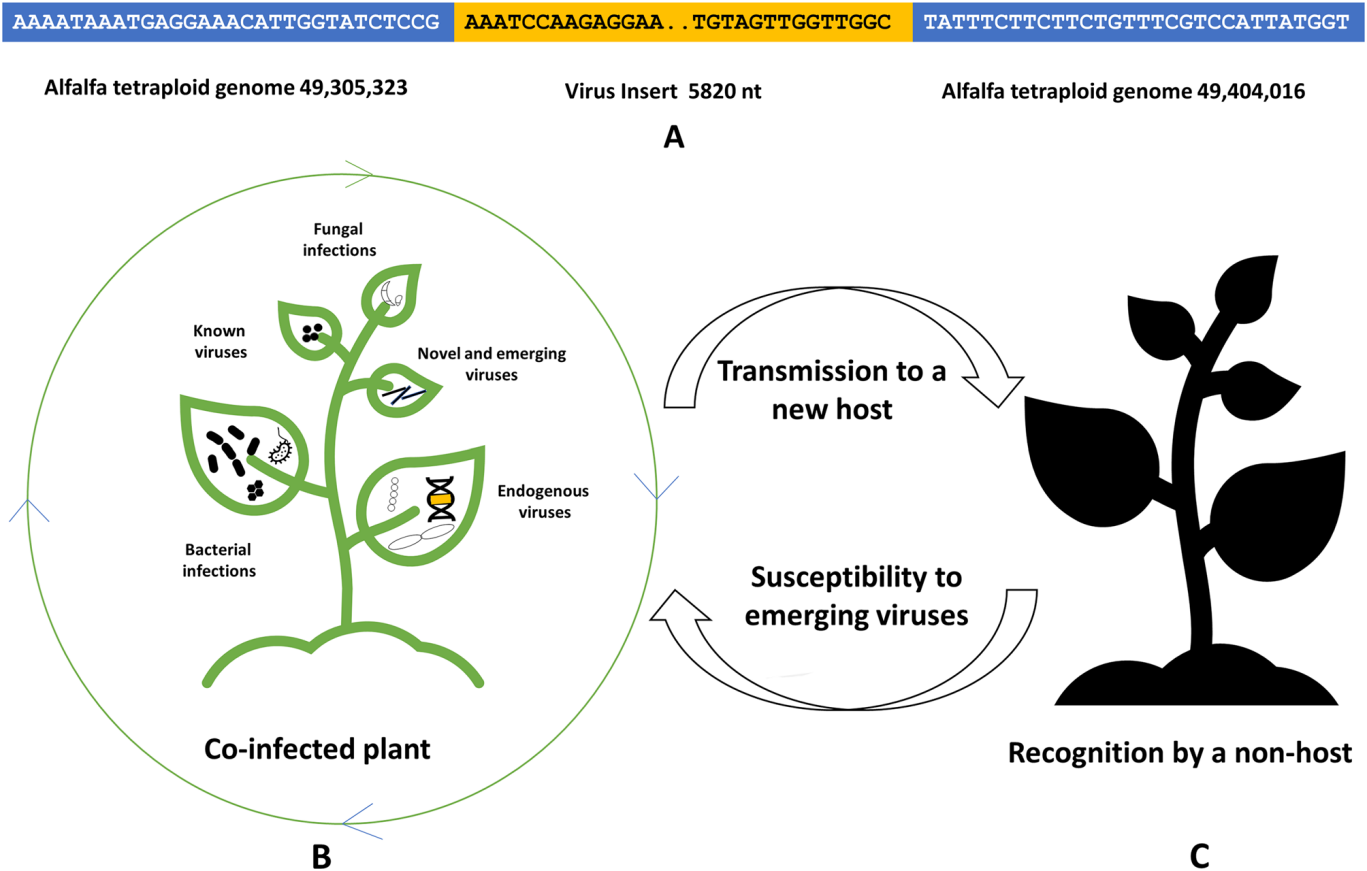
Example of the viral insert in chromosome 8.3 of the tetraploid alfalfa genome (**A**) and a simplified representation of the evolution of pathogen virulence and reduction of host fitness in co-infected plants (**B**,**C**). (**A**) Blue rectangular boxes: portions of alfalfa tetraploid genomes surrounding the viral insert. Yellow rectangular box: 820 nucleotides-long viral insert. Dots indicate continuous nucleotide sequence. (**B**) Multi-pathogen interactions of different types may increase genetic diversity of microbes, result in novel recombinant genotypes, and impact host susceptibility and defense responses. (**C**) Consequently, transmission rates may change, following by host range expansion.

To further confirm that SbCMVL segments identified in this study represent EVEs rather than an exogenous infection, the 6553 nt-long SbCMVL fragment was BLAST searched against the tetraploid alfalfa genome. Table S4 shows distribution of the viral genomic fragments in the tetraploid genome, thus verifying that SbCMVL is an endogenous, integrated virus. Figure 7A illustrates the location of one of the SbCMVL segments in the chromosome 8.3 of the alfalfa tetraploid genome.

When we mapped the longest FMV fragment (892 nt) from the transcriptome assembly against the tetraploid alfalfa genome, up to 228 bp matched the genome (Table S4), assuming that this virus could be of endogenous nature as well, as suggested earlier^26^.

### Contaminant viruses

Contaminant viruses, likely originating from different hosts in the same ecosystem—mostly insects—represented a substantial portion of the findings (Table S5). They were usually detected in one, maximum two alfalfa samples and had very few sequencing reads coupled with them, implying an incidental contamination. Nevertheless, these contaminant viruses are listed in the study because they were associated with alfalfa samples. Besides, due to the frequently low percentage identity and query coverage values in BLAST outputs, a possibility of these viruses being novel species, potentially infecting alfalfa, cannot be excluded.

### Non-viral organisms as part of the multi-pathogenic complexes

Reads originating from non-viral infections were deduced through assessment of the metagenomic data by MEGAN/Diamond BLAST and Kracken2 programs^27,28^. According to this analysis, many alfalfa samples infected with viruses, also contained described bacterial pathogens, including *Clavibacter michiganensis*, a causal agent of bacterial wilt; *Xanthomonas campestris*, a causal agent of bacterial leaf spot; *Xylella fastidosa*, a causal agent of alfalfa dwarf disease; *Agrobacterium tumefaciens*, a causal agent of crown gall disease. In addition, several species in the genus *Pseudomonas*, namely *P. viridiflava*, *P. syringae*, and *P. sevastanoi* were identified, which have been associated with bacterial stem blight of alfalfa (Table S6).

The metagenomic analysis showed that alfalfa samples were co-infected with fungal pathogens as well. Some of these identified included *Alternaria alternata,* a fungus that was reported to cause leaf spot and blight symptoms on alfalfa in Canada^29^ but to the best of our knowledge, not previously reported in U.S. Other fungal species identified included *Stemphylium* spp., causal organism of leaf spot in alfalfa; *Aspergillus* spp. fungi that grow and sporulate in hay; *Fusarium* spp., associated with alfalfa wilt disease; *Colletotrichum* spp., causing alfalfa anthracnose; and *Bipolaris* spp. known to cause alfalfa root rot disease in China^30^ but to the best of our knowledge, not previously reported in the U.S. (Table S5).

## Discussion

Among numerous pathogens that infect alfalfa, viruses are the least recognized members^1^. Recent discoveries, however, contested a widespread view on low economic impact of viral diseases in alfalfa, proposing their essential contribution to the severity of complex infections involving multiple pathogens^1,2,4–9^. In this study, we have investigated a diversity of viral populations in several research and commercial alfalfa fields located in the U.S. states of Washington and Idaho. The following conclusions could be drawn based on the outcome of this work:

1. The prevailing viruses isolated from alfalfa samples in the surveyed areas included both well-known as well as emerging, newly discovered agents. In the first group we described AMV, PeSV, BLR and alfalfa latent virus, a strain of PeSV^1^. The second group contained viruses that were only recently reported in the host and in this country and included alfalfa virus S, lucerne transient streak virus, partitiviruses and amalgavirus.
2. Alfalfa may potentially be a host for viruses that were not previously reported to infect the plant, such as CVT-A. While detection of CVT-A strain is most likely due to the presence of authentic infection, occurrence of HLV and PVX, despite RT-PCR evidence, could be the result of external contamination of alfalfa plants via aphids (HLV) or contaminated equipment (PVX). Negative results of *N. benthamiana* inoculation with alfalfa samples containing PVX reads support this hypothesis. It is noteworthy however, that PVX, among other viruses, was believed to infect clovers (*Trifolium* spp.) and alfalfa in Illinois, according to the plant disease report from the University of Illinois^31^.
3. Several conceivably novel viruses were identified in the survey, including three different rhabdoviruses, a toti-like virus, two picorna-like viruses, and a deltapartitivirus (Table 2). Their exact origin and importance for alfalfa health remains to be clarified, although rhabdoviruses, such as alfalfa dwarf virus, can cause a severe disease in alfalfa^32^. However, it is not known whether these viruses are new to the U.S. The fact that they have been identified in this study may indicate that they are widespread in the surveyed regions and possibly have always been present but gone unnoticed. More information is needed to determine the biological significance of these described viruses.
4. Each tested sample was co-infected with a cluster of different viruses. Occurrence of multiple viral infections can affect within-plant virulence, disease dynamics, host–pathogen interactions, and as a result, trigger epidemiological implications^33^.
5. In many cases, alfalfa samples analyzed in this study were co-infected with viruses and non-viral pathogens, such as bacteria and fungi, suggesting that viruses represent a ubiquitous and abundant background for all other host–pathogen interactions. This sophisticated multi-pathogenic habitat involving viruses as an essential part of the disease complexes can affect behavior of all co-infecting organisms, their intra-host accumulation, and transmission rates^34,35^. Consequently, host fitness may be altered triggering susceptibility, expanding disease damage to the plant and, potentially, disease epidemiology (Fig. 7B,C).
6. This study confirms that alfalfa genome carries integrated pararetrovirus sequences resembling SbCMV. Presumably, the same could be true for FMV. The SbCMV-like virus appears to be the same as earlier identified AePV^26^. Since the integrated segments contain ORFs, which are likely translated into proteins, it is feasible that the viral proteins might have adapted some biological roles in normal growth and development for alfalfa. It is also possible that contribution of EVEs sequences to the crop’s physiology and evolution occurs at the level of gene regulation and/or via supply of new genetic material^36^.

## Methods

### Plant materials

All experiments involving plant materials comply with relevant institutional, national, and international guidelines and legislations. Alfalfa plant samples originated from two U.S. Pacific Northwestern alfalfa producing states. Authors have intentionally omitted specific collection site details to protect producer privacy. In each of the 24 fields involved in the study, ten plants were sampled in a zigzag pattern by collecting the upper 10–15 cm of stems with leaves. During sampling, plants with symptoms associated with possible viral infection were targeted. Plants were represented by commercial varieties (conventional and transgenic) adapted to growing in the areas.

### RNA extraction, HTS and sequencing

Ten samples collected from 10 different plants in each of the 24 fields were split in two to three groups, 3–5 plants in the group. In each group, three to five leaves were taken from each plant, pooled together, and used for total RNA extraction. Total RNA was extracted with TRIzol Reagent (Thermo Fisher Scientific, Waltham, MA USA). cDNA libraries were prepared from total RNA using poly (A) selection protocol for higher exonic coverage and better accuracy of gene quantification^37^. High-throughput sequencing of paired-end reads (2 × 150) was performed on a HiSeq Illumina Platform by Novogene (Sacramento, CA USA).

### Bioinformatic analysis

The reads were cleaned using BBMAP (https://jgi.doe.gov/data-and-tools/software-tools/bbtools/) to remove low-quality reads and adapters sequences. The cleaned reads were mapped to the alfalfa genome and the unmapped reads were used for further analysis. Clean reads were assembled de novo using CLC Genomics Workbench (v. 20.0; Qiagen, Redwood City, CA USA) and mapped to the reference viral genomes, when available, using CLS’s maximal exact match algorithm or Bowtie 2 aligner^38^. Other assemblies were created from the complete set of reads for each sample after trimming with Trimmomatic^39^ and then running BLASTX against a custom viral detection database containing all viral sequences in NCBI refseq (https://www.ncbi.nlm.nih.gov/genome/viruses/), viruses that have not been deposited in refseq yet, plus *Arabidopsis lyrata* and *A. thaliana* proteins added from the reference proteome of UniProt for an in-BLAST subtraction.

Contig matches and relatives to known viruses were then mapped in CLC Genomics Workbench (minimum 80% identity for 50% of read length); contigs were extracted and read counts and coverages were generated for each sample. For identification of the endogenous viruses, the 6.5 kb putative soybean chlorotic mottle virus-like (SbCMVL) and 892 nt figwort mosaic virus-like (FMVL) transcripts identified in the alfalfa transcriptome were searched using the BLASTN program against the tetraploid alfalfa genome^25^. The BLAST hits were parsed for sequence identity, hsp (high-scoring pair) hit-region and expect-value.

Contigs derived from bacteria and fungi were identified by using the MEGAN/Diamond BLAST and Kracken2 programs^27,28^. Paired-end reads were merged using BBmerge^40^ and both merged and unmerged single-end reads were concatenated, followed by BLASTX screen against the nr database using the DIAMOND BLAST program (v2.0.6.144). The BLAST results were meganized^27^ and inputted into MEGAN6 for taxonomic identification. In addition, paired-end reads were inputted into the Kracken2 (v2.1.1) pipeline using the standard Kracken database for taxonomic identification. Relevant taxa were then checked manually using the BLASTN program against nr at the NCBI.

### RT-PCR and Sanger sequencing

Additional sequence information on a select group of genomes was obtained by SMARTer 5′/3′ RACE (Takara Bio, Mountain View, CA USA), cloning and sequencing of the multiple RT-PCR products. RT-PCRs were performed with total RNA employing the SuperScript RT-PCR system per the manufacturer’s directions (Thermo Fisher Scientific, Waltman, MA USA). Gel-purified RT-PCR products were either sequenced directly or cloned into the pCRII-TOPO vector with dual promoter (Thermo Fisher Scientific, Waltman, MA USA) for an automated Sanger sequencing.

### Phylogenetic assignments of novel contigs

Phylogenetic trees were deduced from CLUSTALW alignments and built using MEGA 7 software^41^ with Maximum Likelihood method based on the Tamura-Nei model and bootstrap analysis of 1000 replicates.

## Supplementary Information

Supplementary Information 1.

Supplementary Information 2.

Supplementary Information 3.

Supplementary Information 4.

Supplementary Information 5.

Supplementary Information 6.

Supplementary Information 7.

## Data Availability

The datasets for this study are available at the NCBI’s BioProject PRJNA768336, Submission SUB10460191; and accessions OK181162, OK181163, OK514705, OK514706, OK514707, OK514708, OK514709, and OK514710. Other relevant data are within the manuscript and its supplementary files.

## References

[1] Bejerman N, Roumagnac P, Nemchinov LG (2020). High-throughput sequencing for deciphering the virome of alfalfa (*Medicago sativa* L.). Front. Microbiol..

[2] Bejerman N (2011). First report of a rhabdovirus infecting alfalfa in Argentina. Plant Dis..

[3] Bejerman N (2015). Complete genome sequence and integrated protein localization and interaction map for alfalfa dwarf virus, which combines properties of both cytoplasmic and nuclear plant rhabdoviruses. Virology.

[4] Roumagnac P (2015). Alfalfa leaf curl virus: An aphid-transmitted geminivirus. J. Virol..

[5] Nemchinov LG, Grinstead SC, Mollov DS (2017). Alfalfa virus S, a new species in the family Alphaflexiviridae. PLoS ONE.

[6] Nemchinov LG (2018). Characterization of alfalfa virus F, a new member of the genus *Marafivirus*. PLoS ONE.

[7] Gaafar YZA (2019). Characterization of a novel nucleorhabdovirus infecting alfalfa (*Medicago sativa*). Virol J..

[8] Nemchinov LG, Grinstead S (2020). Identification of a novel isolate of alfalfa virus S from China suggests a possible role of seed contamination in the distribution of the virus. Plant Dis..

[9] Samarfard S, McTaggart AR, Sharman M, Bejerman NE, Dietzgen RG (2020). Viromes of ten alfalfa plants in Australia reveal diverse known viruses and a novel RNA virus. Pathogens.

[10] Samac DA, Rhodes LH, Lamp WO (2016). Compendium of alfalfa diseases and pests.

[11] Jiang P, Shao J, Nemchinov LG (2019). Identification of emerging viral genomes in transcriptomic datasets of alfalfa (*Medicago sativa* L.). Virol. J..

[12] Moreno AB, López-Moya JJ (2020). When viruses play team sports: Mixed infections in plants. Phytopathology.

[13] Milusheva S (2019). Molecular analysis of the complete genome of an unusual virus detected in sweet cherry (*Prunus avium*) in Bulgaria. Eur. J. Plant Pathol..

[14] Blackstock JM (1978). Lucerne transient streak and Lucerne latent, two new viruses of lucerne. Aust. J. Agric. Res..

[15] Kim H, Park D, Hahn Y (2018). Identification of novel RNA viruses in alfalfa (*Medicago sativa*): an alphapartitivirus, deltapartitivirus, and a marafivirus. Gene.

[16] Thekke-Veetil T (2020). Soybean thrips (Thysanoptera: *Thripidae*) harbor highly diverse populations of arthropod, fungal and plant viruses. Viruses.

[17] Forster LS, Jones T (1979). Properties of lucerne transient streak virus, and evidence of its affinity to southern bean mosaic virus. Ann. Appl. Biol..

[18] Forster, L. S. & Jones, T. Lucerne transient streak virus. Descriptions of plant viruses. DPV №: 224. https://www.dpvweb.net/dpv/showdpv/?dpvno=224 (1980).

[19] Wickner RB, Ghabrial SA, Nibert ML, Patterson JL, Wang CC, King AMO, Adams MJ, Carstens EB, Lefkowitz EJ (2012). Family *Totiviridae*. Virus Taxonomy, Ninth Report of the International Committee on Taxonomy of Viruses.

[20] Shi M (2016). Redefining the invertebrate virosphere. Nature.

[21] Harvey E (2019). Identification of diverse arthropod associated viruses in native Australian fleas. Virology.

[22] François S (2021). Characterization of the viral community associated with the alfalfa weevil (*Hypera postica*) and its host plant, alfalfa (*Medicago sativa*). Viruses.

[23] Saqib M, Wylie SJ, Jones MGK (2015). Serendipitous identification of a new Iflavirus-like virus infecting tomato and its subsequent characterization. Plant Pathol..

[24] Osaki H, Sasaki A, Nakazono-Nagaoka E, Ota N, Nakaune R (2017). Genome segments encoding capsid protein-like variants of Pyrus Pyrifolia Cryptic Virus. Virus Res..

[25] Chen H (2020). Allele-aware chromosome-level genome assembly and efficient transgene-free genome editing for the autotetraploid cultivated alfalfa. Nat Commun..

[26] Boutanaev AM, Nemchinov LG (2021). Genome-wide identification of endogenous viral sequences in alfalfa (*Medicago sativa* L.). Virol. J..

[27] Bağcı C, SaschaPatz S, Huson DH (2021). DIAMOND+MEGAN: Fast and easy taxonomic and functional analysis of short and long microbiome sequences. Curr. Protoc..

[28] Wood DE, Lu J, Langmead B (2019). Improved metagenomic analysis with Kraken 2 (2019). Genome Biol..

[29] Abbasi PA, Ali S, Renderos W, Naeem HA, Papadopoulos Y (2018). First report of *Alternaria alternata* causing leaf spot and blight symptoms on alfalfa in Canada. Can. J. Plant Pathol..

[30] Li YG (2019). Occurrence of bipolaris root rot caused by *Bipolaris sorokiniana* on alfalfa in China. Plant Dis..

[31] Babadoost, M. Virus diseases of alfalfa and clovers in Illinois. *Reports on Plant Diseases,* RPD No. 307. University of Illinois Extension, University of Illinois at Urbana-Champaign. http://ipm.illinois.edu/diseases/series300/rpd307/ (1990).

[32] Dietzgen RG (2020). Diversity and epidemiology of plant rhabdoviruses. Virus Res..

[33] Tollenaere C, Susi H, Laine AL (2016). Evolutionary and epidemiological implications of multiple infection in plants. Trends Plant Sci..

[34] Abdullah AS (2017). Host–multi-pathogen warfare: pathogen interactions in co-infected plants. Front. Plant Sci..

[35] Dutt A, Didier Andrivon D, Christophe LeMay C (2022). Multi-infections, competitive interactions, and pathogen coexistence. Plant Pathol..

[36] Geering ADW (2014). Endogenous florendoviruses are major components of plant genomes and hallmarks of virus evolution. Nat. Commun..

[37] Zhao S, Zhang Y, Gamini R, Zhang B, von Schack D (2018). Evaluation of two main RNA-seq approaches for gene quantification in clinical RNA sequencing: PolyA+ selection versus rRNA depletion. Sci. Rep..

[38] Langmead B, Salzberg S (2012). Fast gapped-read alignment with Bowtie 2. Nat. Methods.

[39] Bolger, A. M., Lohse, M., & Usadel, B. Trimmomatic: A flexible trimmer for Illumina Sequence Data. *Bioinformatics*, btu170 (2014). 10.1093/bioinformatics/btu170 PMC4103590 24695404

[40] Bushnell B, Rood J, Singer E (2017). BBMerge—Accurate paired shotgun read merging via overlap. PLoS ONE.

[41] Kumar S, Stecher G, Tamura K (2016). MEGA7: Molecular evolutionary genetics analysis version 70 for bigger datasets. Mol. Biol. Evol..

