# Supplementary material for: Diversity of the virome associated with alfalfa (Medicago sativa L.) in the U.S. Pacific Northwest
Source: Sci Rep. 2022 May 24;12:8726. doi: 10.1038/s41598-022-12802-4 (PMC9130302; doi:10.1038/s41598-022-12802-4)
Supplement: Supplementary file 1 — Supplementary Information 1. [file 41598_2022_12802_MOESM1_ESM.pdf]

Supplementary Figure 1

Original full-length images of the gels shown in Figure 3 of the main text.

(These images were cropped to combine and fit them in one figure)

Figure 3A. RT-PCR detection of cherry virus Trakiya, alfalfa strain

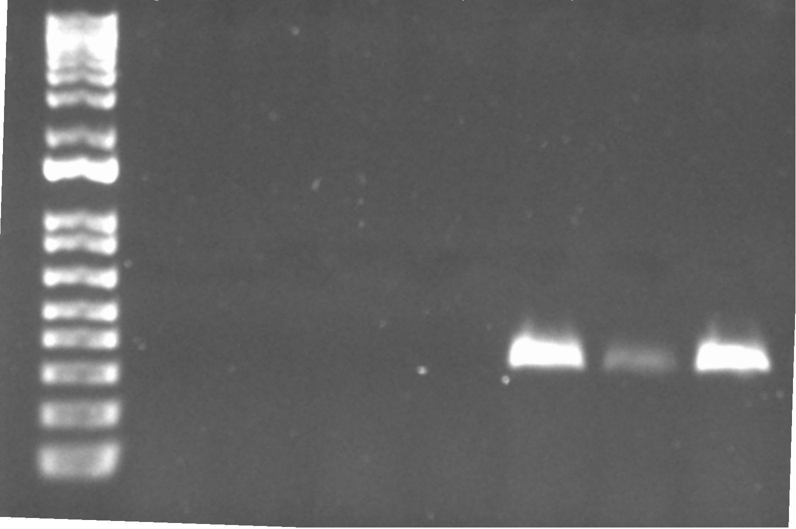

Figure 3B. RT-PCR detection of hop latent virus

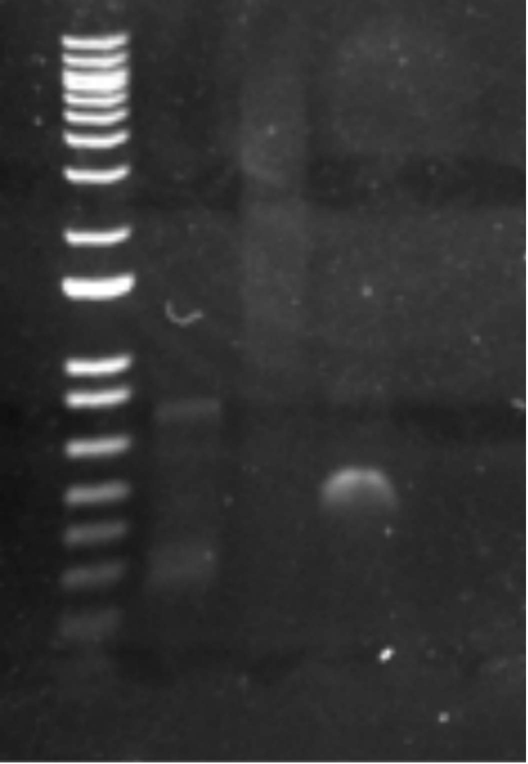

Figure 3C. RT-PCR detection of potato virus X

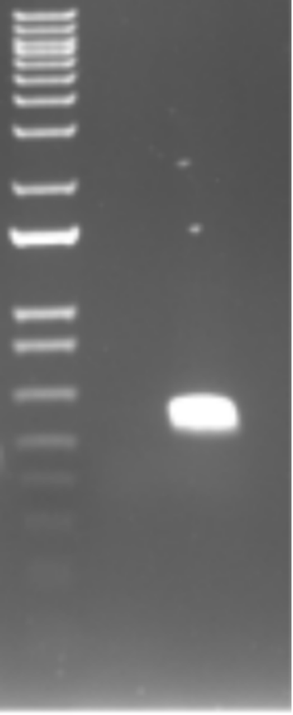

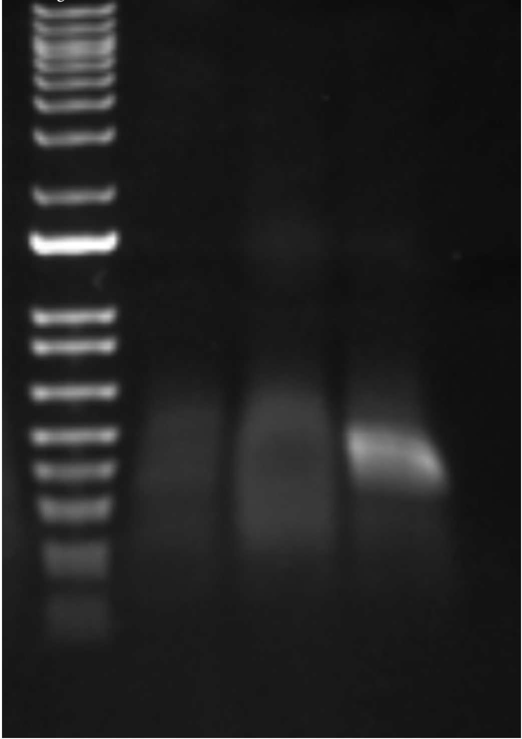

Figure 3D. RT-PCR detection of alfalfa nucleorhabdovirus 1
