# Supplementary material for: Diversity of the virome associated with alfalfa (Medicago sativa L.) in the U.S. Pacific Northwest
Source: Sci Rep. 2022 May 24;12:8726. doi: 10.1038/s41598-022-12802-4 (PMC9130302; doi:10.1038/s41598-022-12802-4)
Supplement: Supplementary file 6 — Supplementary Information 6. [file 41598_2022_12802_MOESM6_ESM.pdf]

Supplementary Table 5

Putative contaminant viruses

**Virus name**	**Host**
Ancient Northwest Territories cripavirus	Mammals
Aphis glycines virus 1	Soybean aphid
Bombus-associated virus Pic2	Bumblebee
Bundaberg bee virus 8	Honeybee
Diabrotica virgifera virgifera virus 2	Western Corn Rootworm
Faecal-associated gemycircularvirus	Mammals, birds
Hubei toti-like virus 2	Arthropoda
HVAC-associated RNA virus 1	Indoor Air
Kilifi Virus	Drosophilidae
La Jolla virus	Drosophila melanogaster
Lasius niger virus 1	Black garden ant
Luckshill virus	Drosophila suzukii
Neofusicoccum parvum narnavirus 1	Phytopathogenic fungus
Nesidiocoris tenuis iflavirus 1	Hemiptera
River Liunaeg virus	Bumblebee
Solenopsis invicta virus 7	Fire ant
